# Resolved phylogeny and biogeography of the root pathogen *Armillaria* and its gasteroid relative, *Guyanagaster*

**DOI:** 10.1186/s12862-017-0877-3

**Published:** 2017-01-25

**Authors:** Rachel A. Koch, Andrew W. Wilson, Olivier Séné, Terry W. Henkel, M. Catherine Aime

**Affiliations:** 1 Department of Botany and Plant Pathology, Purdue University, West Lafayette, IN 47907 USA; 2 Sam Mitchel Herbarium of Fungi, Denver Botanic Gardens, Denver, CO 80206 USA; 3 Institute of Agricultural Research for Development (IRAD), National Herbarium of Cameroon (MINRESI), PO Box 1601, Yaoundé, Cameroon; 4 Department of Biological Sciences, Humboldt State University, Arcata, CA 95521 USA

**Keywords:** Armillaria root rot, Cameroon, Eocene, Fungal taxonomy, Gasteromycetation, Guiana Shield, Melanin, Mushroom evolution, Physalacriaceae, Systematics

## Abstract

**Background:**

*Armillaria* is a globally distributed mushroom-forming genus composed primarily of plant pathogens. Species in this genus are prolific producers of rhizomorphs, or vegetative structures, which, when found, are often associated with infection. Because of their importance as plant pathogens, understanding the evolutionary origins of this genus and how it gained a worldwide distribution is of interest. The first gasteroid fungus with close affinities to *Armillaria*—*Guyanagaster necrorhizus*—was described from the Neotropical rainforests of Guyana*.* In this study, we conducted phylogenetic analyses to fully resolve the relationship of *G. necrorhizus* with *Armillaria*. Data sets containing *Guyanagaster* from two collecting localities, along with a global sampling of 21 *Armillaria* species—including newly collected specimens from Guyana and Africa—at six loci (28S, *EF1α, RPB2, TUB, actin-1* and *gpd*) were used. Three loci—28S, *EF1α* and *RPB2*—were analyzed in a partitioned nucleotide data set to infer divergence dates and ancestral range estimations for well-supported, monophyletic lineages.

**Results:**

The six-locus phylogenetic analysis resolves *Guyanagaster* as the earliest diverging lineage in the armillarioid clade. The next lineage to diverge is that composed of species in *Armillaria* subgenus *Desarmillaria*. This subgenus is elevated to genus level to accommodate the exannulate mushroom-forming armillarioid species. The final lineage to diverge is that composed of annulate mushroom-forming armillarioid species, in what is now *Armillaria sensu stricto.* The molecular clock analysis and ancestral range estimation suggest the most recent common ancestor to the armillarioid lineage arose 51 million years ago in Eurasia. A new species, *Guyanagaster lucianii* sp. nov. from Guyana, is described.

**Conclusions:**

The armillarioid lineage evolved in Eurasia during the height of tropical rainforest expansion about 51 million years ago, a time marked by a warm and wet global climate. Species of *Guyanagaster* and *Desarmillaria* represent extant taxa of these early diverging lineages. *Desarmillaria* represents an armillarioid lineage that was likely much more widespread in the past. *Guyanagaster* likely evolved from a gilled mushroom ancestor and could represent a highly specialized endemic in the Guiana Shield. *Armillaria* species represent those that evolved after the shift in climate from warm and tropical to cool and arid during the late Eocene. No species in either *Desarmillaria* or *Guyanagaster* are known to produce melanized rhizomorphs in nature, whereas almost all *Armillaria* species are known to produce them. The production of rhizomorphs is an adaptation to harsh environments, and could be a driver of diversification in *Armillaria* by conferring a competitive advantage to the species that produce them.

**Electronic supplementary material:**

The online version of this article (doi:10.1186/s12862-017-0877-3) contains supplementary material, which is available to authorized users.

## Background

The globally distributed mushroom-forming genus *Armillaria* contains species that are frequently encountered as tree pathogens in natural forests [1, 2] as well as silvicultural and agronomic systems [3, 4], and are the causal agent of the root disease Armillaria root rot [5]. Besides being pathogens, *Armillaria* species also play a critical role as decomposers. Extensive infection observed in stumps and roots and long possession of the substrate suggest that *Armillaria* species contribute significantly to decomposition and mineral cycling within many forests [6].

*Armillaria* species form basidiomes—in this case, mushrooms—which serve as reproductive structures that fruit seasonally when conditions are optimal. Each of these basidiomes produces their reproductive propagules, or basidiospores, on an exposed spore-bearing surface, or hymenium. At maturity, the basidiospores are propelled into the air column via a mechanism of forcible spore discharge known as ballistospory. Basidiospores of other fungi capable of ballistospory have been shown to disperse between continents [7–9]. Based on molecular clock analyses, the origin of *Armillaria* post-dates the Gondwana break-up, leading to the hypothesis that several such long distance basidiospore dispersal events led to the global distribution of *Armillaria* [10].

One change in basidiome form that has occurred repeatedly within mushroom-forming lineages is the shift from producing basidiospores on an exposed hymenium to producing them within an enclosed hymenium. This loss of an exposed hymenium has resulted in the loss of ballistospory [11]. Fungi that undergo this change will hereinafter be referred to as gasteroid fungi as they go through a process called gasteromycetation. A suite of changes in basidiome morphology, including the development of a fully enclosed basidiospore-bearing mass (termed a gleba) that is encased in a specialized covering (termed a peridium), typically take place during gasteromycetation. Gasteromycetation is believed to be a unidirectional process, as gasteroid fungi have never been observed to regain the mechanism of ballistospory [11]. In order to persist, gasteroid fungi have evolved a diverse array of dispersal mechanisms that engage exogenous forces. Examples include animal mycophagy for volatile-producing underground truffle-like basidiomycetes [12] and the “bellows” mechanism of puffballs [13].

Gasteroid fungi have evolved independently many times from ballistosporic ancestors within the Agaricomycetidae (e.g. [14–20]). These ancestors of gasteroid fungi include agaricoid lineages (i.e. those with lamellate hymenophores in the Agaricales and Russulales) and boletoid lineages (i.e. those with porose-tubulose hymenophores in the Boletales). To date, gasteroid fungi have only been documented as ectomycorrhizal (ECM) or saprotrophic in their ecology (e.g. [11, 21–26]).

The first known gasteroid fungus closely related to *Armillaria* was described in 2010 [27]. The monotypic genus, *Guyanagaster* T. W. Henkel, Aime & M. E. Sm. and the species *G. necrorhizus* T. W. Henkel, Aime & M. E. Sm. (Basidiomycota; Agaricales; Physalacriaceae) are only known from the Pakaraima Mountains of Guyana in the Guiana Shield region of South America [27]. This fungus produces subhypogeous basidiomes that are often attached to decaying tree roots, has a thick black peridium with pyramidal warts, a reduced stipe, and a tough, gelatinous gleba that changes from white to pink to brick red with maturation [27]. This species is remarkable in the sense that it does not possess the morphological hallmarks of gasteroid fungi adapted for any of the aforementioned dispersal mechanisms (e.g. mammal or mechanical dispersal).

To our knowledge, until the discovery of *Guyanagaster*, no known gasteroid fungus has been demonstrated to have evolved from a pathogenic lineage. *Guyanagaster necrorhizus* basidiomes are almost always found fruiting from dead and decaying tree roots, in this case primarily of the forest-dominating trees in the genus *Dicymbe* Spruce ex Benth. (Fabaceae subfam. Caesalpinioideae). Roots to which *G. necrorhizus* are attached display signs of white rot, indicating a wood decay capability for this fungus. Given the association of *G. necrorhizus* to dead and decaying roots and its close relationship to *Armillaria* species, it is conjectured that it is also has pathogenic capabilities, but no experimental evidence exists to confirm this.

Prior multi-gene phylogenetic analyses by Henkel et al. were equivocal in that some loci suggested *G. necrorhizus* was sister to *Armillaria*, while other loci showed *G. necrorhizus* derived from within *Armillaria* [27]. Resolving the phylogenetic placement of *G. necrorhizus* in relation to *Armillaria* will create a more complete picture of armillariod (a term to denote both *Armillaria* and *Guyanagaster* species) evolution through time and allow us to understand traits that led to the success of this group. A resolved phylogeny will also give us a robust framework in which to understand the evolution of *Guyanagaster*.

Here we use molecular data from *G. necrorhizus* and *Armillaria* species from every major infrageneric lineage, including *Armillaria puiggarii* Speg.—the only *Armillaria* species to be collected in the same region as *G. necrorhizus*—to provide a fully resolved phylogenetic hypothesis for armillarioid fungi*.* We also erect a new genus, *Desarmillaria*, composed of the exannulate mushroom-forming armillarioid species, to accommodate those species formerly placed in *Armillaria* subgenus *Desarmillaria.* A second species of *Guyanagaster*, *Guyanagaster lucianii,* is described as new to science. Using these data we produce a time-calibrated phylogeny, which we analyze in combination with ancestral range estimations, in order to examine the morphological and biogeographic evolution of armillarioid fungi.

## Methods

### Collecting and morphological analyses

Collecting expeditions to the Upper Potaro River Basin (hereafter referred to as the Potaro) in the west-central Pakaraima Mountains of Guyana were conducted during the rainy seasons of May–July of 2002–2013. Fungi were collected within a 15-km radius of a previously established base camp (5°18'04.80"N, 59°54'40.40"W) in forests dominated by *Dicymbe corymbosa* Spruce ex Benth. and *Dicymbe altsonii* Sandw. (Fabaceae subfam. Caesalpinioideae) [28, 29]. In December 2013–January 2014, a collecting expedition to the Mabura Ecological Reserve (hereafter referred to as Mabura) in Mabura Hill, Guyana—approximately 125 km from the Potaro—was conducted. Fungi were collected within a 3-km radius of the field station (5°10'09.26"N, 58°42'14.40"W) in forests with high densities of *D. altsonii* [30]. New collections of African *Armillaria* species were made in the Dja Biosphere Reserve in the East Province of Cameroon during the rainy season of August–September 2014 within a 2-km radius of the Dja base camp (3°21'29.80"N, 12°43'46.90"W).

Fresh morphological characteristics and substratum relationships were described in the field. Color was described subjectively and coded according to Kornerup and Wanscher [31], with color plates noted in parentheses. The descriptions of the collected *Armillaria* specimens were compared to the descriptions of validly published species. Additionally, attempts to obtain specimen cultures were made in the field by placing small pieces of unexposed basidiome tissue on Potato Dextrose Agar (PDA) (BD Difco™, Franklin Lakes, New Jersey, USA) and grown for one month at room temperature. Specimens were then field-dried with silica gel.

Micromorphological features on dried specimens were examined in the laboratory using an Olympus BH2-RFCA compound microscope. Two dried *Armillaria* specimens from Africa, TH 9926 and THDJA 91, and four dried *Guyanagaster* specimens, MCA 4424, RAK 84, RAK 88 and RAK 89, were rehydrated in 70% ethanol and then sectioned by hand and mounted in water, Melzer’s reagent and 1% aqueous Congo red. Twenty randomly selected basidiospores were measured per collection under a 100× objective. Length/width Q values for basidiospores are reported as Q_r_ (range of Q values over 20 basidiospores measured) and Q_m_ (mean of Q values ± SD). Specimens were deposited in the following herbaria: PUL (Kriebel Herbarium), BRG (Guyana National Herbarium), HSC (Humboldt State University) and YA (National Herbarium of Cameroon).

### Molecular methods

DNA was extracted from basidiome tissue using the Wizard® Genomic DNA Purification kit (Promega Co., Madison, Wisconsin, USA). Specimens housed in the Kriebel Herbarium at Purdue University were used to obtain DNA if sequence data for equivalent taxa were not available on the NCBI Nucleotide database. PCR reactions included 12.5 μL of MeanGreen 2× Taq DNA Polymerase PCR Master Mix (Syzygy Biotech, Grand Rapids, Michigan, USA), 1.25 μL of each primer (at 10 μM) and approximately 100 ng of DNA. The final PCR reaction volume was 25 μL. The recommended cycling conditions for each primer pair we used were followed.

To determine the identity of the recently collected *Armillaria* specimens from Guyana and Africa, as well as to confirm the identity of the recently collected *Guyanagaster* specimens, PCR was performed to acquire sequence data from the internal transcribed spacer (ITS) region (inclusive of ITS1, 5.8S and ITS2 regions), using the primer pair ITS1F/ITS4B [32]. To analyze the phylogenetic relationship of the armillarioid fungi, PCR was performed on all specimens at the three following loci: nuclear ribosomal large subunit DNA (28S), *Elongation Factor 1-α* (*EF1α*) and *RNA polymerase II* (*RPB2*) genes using the following primer pairs, respectively: LROR/LR6 [33, 34], 987 F/2218R [35] and bRPB2-6 F/bRPB2-7.1R [36].

Uncleaned PCR products were sent to Beckman Coulter, Inc. (Danvers, Massachusetts, USA) for sequencing. Sequences were manually edited using Sequencher 5.2.3 (Gene Codes Corporation, Ann Arbor, Michigan, USA). The ITS sequences we generated were used as queries in the sequence similarity search tool, NCBI BLAST, to search the NCBI Nucleotide database.

### Phylogenetic analyses

In order to resolve the phylogeny of *Guyanagaster* and *Armillaria*, we compiled a dataset composed of sequence data from both *Armillaria* and *Guyanagaster* specimens. Two closely related species in the Physalacriaceae, *Oudemansiella mucida* and *Strobilurus esculentus,* served as outgroup taxa. Six loci were used (28S, *EF1α*, *RPB2*, *Actin-1* (*actin-1*), *Glyceraldehyde-3-Phosphate Dehydrogenase* (*gpd*) and *Beta-Tubulin* (*TUB*)) to assess *Guyanagaster* and *Armillaria* systematic relationships through phylogenetic analysis. Not all loci were available for all specimens. DNA sequences that were not generated in this study were obtained from GenBank or published whole genome sequences. Collection information for all specimens and GenBank accession numbers for the included sequences are compiled in Additional file 1.

Sequences were aligned in Mega 5.0 [37] using the MUSCLE algorithm [38] with refinements to the alignment done manually. The introns of *EF1α* were alignable across the *Armillaria*/*Guyanagaster* dataset and were therefore included. Phylogenies were reconstructed using maximum likelihood (ML) and Bayesian methods. The GTR + G model of molecular evolution was selected for all data sets as determined by PartitionFinder v1.1.0 [39]. Maximum likelihood bootstrap analysis for phylogeny and assessment of the branch support by bootstrap percentages (BS%) was performed using RAxML v2.2.3 [40]. One thousand bootstrap replicates were produced. Each locus was analyzed separately as well as all together in a supermatrix data set using ML. Bayesian analyses for the reporting of Bayesian posterior probability (BPP) support for branches was conducted on individual loci as well as the six-gene data set using the program Mr. Bayes v3.2.2 [41]. Four simultaneous, independent runs each with four Markov chain Monte Carlo (MCMC) chains, were initiated and run at a temperature of 0.15 for 20 million generations, sampling trees every 1000 generations until the standard deviation of the split frequencies reached a final stop value of 0.01. We discarded the initial 10% of trees as burn in and produced a maximum clade credibility tree from the remaining trees; 0.95 BPP represents a well-supported lineage.

### Divergence time estimation

Divergence ages within the armillarioid clade were estimated using the fossil calibration approach described and implemented by [24, 42–44]. Molecular clock analysis was performed using BEAST v1.8.3 [45] with XML files containing BEAST commands and priors assembled in BEAUTi v1.8.3. These files included the following analytical settings: GTR + G model of evolution, uncorrelated relaxed clock with lognormal rate distribution; tree prior was set to speciation birth-death process, running 100 million generations, sampling every 1000^th^ tree. The analysis was run three times. The first 10% of trees were removed as burn-in after ensuring a minimum effective sample size (ESS) of 200 was reached for all of the parameters. The remaining trees—representing the posterior distribution from all Bayesian analyses—were combined into a single file using LogCombiner v1.8.3. This file was used to produce a summary tree using Tree Annotator v1.8.3. The mean ages of supported nodes, as well as the corresponding 95% highest posterior densities (HPDs), were examined from BEAST logfiles using Tracer v1.6 [46].

Taxa used in this analysis are in bold in Additional file 1. It includes 21 *Armillaria* species (one representative specimen from each of the species in the first analysis), two *Guyanagaster* specimens (one representative from each collecting locality), nine Physalacriaceae species (one representative from nine different genera that encompass the major lineages within this family fide [47]), while six species from Agaricales families closely related to the Physalacriaceae fide [48] served as outgroup taxa. The alignment was analyzed using five partitions: 28S was analyzed in a single partition, while two codon partitions ((1 + 2), 3) were utilized for both *EF1α* and *RPB2*. The marasmioid fungi (*Marasmius alliaceus*, *Marasmius rotula* and *Mycena amabilissima*) were calibrated based on a 90-Ma fossil *Archaeomarasmius leggetti* from mid-Cretaceous amber [49], following the parameter settings of [24]. In BEAUTi, this prior was set as a lognormal distribution with a mean of 10, log standard deviation of 1 and offset of 90, with mean in real space, truncated on the lower end at 90 and at the upper end at 200.

### Ancestral range estimation

To estimate the ancestral range of the armillarioid clade and lineages within, we used dispersal-extinction-cladogenesis (DEC) analysis in the package Lagrange [50] and implemented in the program RASP [51]. Six areas were defined: North America, Europe + Asia (hereinafter referred to as Eurasia), Africa, tropical South America (composed of the area covered by tropical rainforest in South America), Australasia (composed of Australia, New Zealand and southeast Asia) and temperate South America (composed of the area not covered by tropical rainforest in South America). Each taxon in our data set was assigned to areas based on its current known range. In the scenario we tested, movement between any area at any time was unconstrained. Under this model, all areas are treated as equally probable ancestral ranges. This model was tested under both two and three area constraints. The consensus tree produced during the divergence time analysis was used for all Lagrange calculations and the root node of the armillarioid clade was calibrated to 51 Ma.

## Results

### Sequences, collections and cultures

Eight ITS, eleven 28S, twelve *EF1α* and five *RPB2* sequences were generated during this study (Additional file 1). The size of the sequences ranged from 514–713, 705–1031, 796–1233, and 553–704 bp, respectively. After the ends of the individual alignments were trimmed, the size of the aligned datasets were as follows: 28S was 906 bp; *EF1α* was 906 bp; *RPB2* was 638 bp; *actin-1* was 607 bp; *gpd* was 499 bp; *TUB* was 911 bp. The six-gene dataset was composed of a total of 52 *Armillaria* specimens, representing 21 species, as well as four *Guyanagaster* specimens, representing two species. The number of taxa in each of the single-locus phylogenies is as follows: 28S had 42 taxa; *EF1α* had 57 taxa; *RPB2* had 32 taxa; *actin-1* had19 taxa; *gpd* had 19 taxa; *TUB* had 12 taxa. Phylogenies for each locus, as well as a combination of loci, are available in Additional file 2.

Two *Armillaria* specimens were collected from Guyana—MCA 3111 and TH 9751. ITS sequences from both shared 99% identity with *Armillaria puiggarii* (GenBank: FJ664608). No significant morphological differences were found when comparing our field descriptions to the species description by [52]. Two *Armillaria* specimens were collected from Cameroon—TH 9926 and TH DJA 91. ITS sequences from both shared 99% identity with an unidentified *Armillaria* species collected from Zimbabwe (GenBank: AY882982). Additionally, there was variation between the two sequences. No significant morphological differences were found when comparing TH 9926 to the species description of *Armillaria camerunensis* [53, 54], but there was some morphological variation when comparing TH DJA 91. For now, we conservatively hypothesize that both of these specimens represent *Armillaria camerunensis*. Morphological descriptions of TH 9926 and TH DJA 91 are available in Additional file 3.

**Fig. 1:**
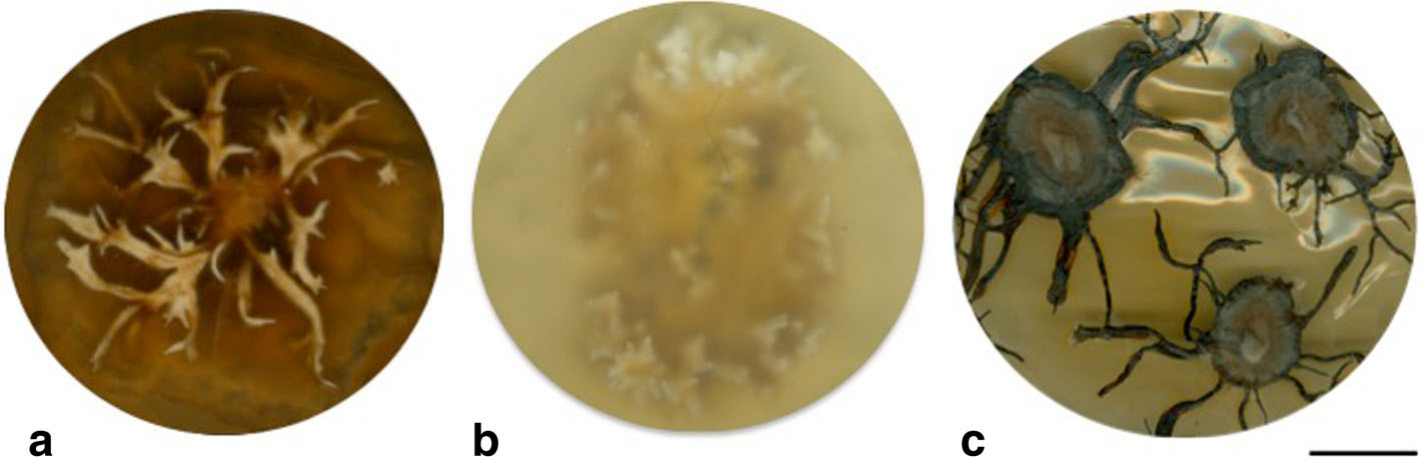
**a** Vegetative culture of *Guyanagaster necrorhizus* MCA 3950 showing unmelanized rhizomorphs. **b** Vegetative culture of *Guyanagaster lucianii* RAK 88 showing unmelanized rhizomorphs. **c** Vegetative culture of *Armillaria puiggarii* TH 9751 showing melanized rhizomorphs. Bar = one cm

Pure vegetative cultures were obtained from *Guyanagaster* specimens MCA 3950 and RAK 88, as well as *Armillaria puiggarii* specimen TH 9751, with rhizomorphs produced in each (Fig. 1). After one month, differences in growth and branching pattern of the rhizomorphs can be observed between the cultures of the two *Guyanagaster* specimens, but the rhizomorphs of neither were observed to become melanized. In contrast, the rhizomorphs in the culture of *A. puiggarii* became melanized almost completely. Strain MCA 3950 is deposited in Centraalbureau voor Schimmelcultures with the strain accession number CBS 138623, while strains RAK 88 and TH 9751 are available from the authors upon request.

### Resolved *Guyanagaster* phylogenetic hypothesis

**Fig. 2:**
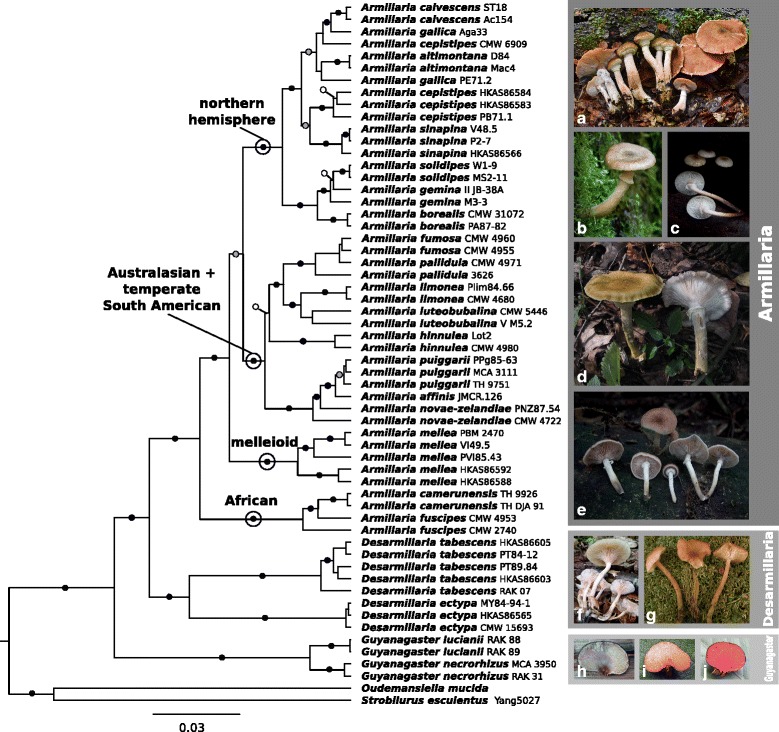
Phylogram generated from the analysis of six gene regions (28S, *EF1α, RPB2, TUB, gpd* and *actin-1*) from 58 taxa. *Guyanagaster* is the earliest diverging lineage and is sister to the mushroom-forming armillarioid species. The exannulate armillarioid species are the next to diverge, and compose *Desarmillaria*. The annulate armillarioid species form a monophyletic lineage and compose *Armillaria sensu stricto*. The major lineages within *Armillaria* are indicated in bold text at the corresponding node. The exannulate armillarioid species are sister to the annulate armillarioid species. *Strobilurus esculentus* and *Oudemansiella mucida* were selected as outgroup taxa. Black circles represent support of 90% (maximum likelihood bootstrap values, shown as percentages) and 0.95 BPP (Bayesian posterior probabilities) or greater, grey circles represent support of 0.95 BPP or greater and white circles represent support of 75% or greater. Images: (**a**) *Armillaria sinapina*; (**b**) *Armillaria hinnulea*; (**c**) *Armillaria puiggarii*; (**d**) *Armillaria mellea*; (**e**) *Armillaria camerunensis*; (**f**) *Desarmillaria tabescens*; (**g**) *Desarmillaria ectypa*, (**h**–**j**) *Guyanagaster necrorhizus*. (Photo credits: (**a**) Christian Schwarz; (**b**) J. J. Harrison; (**c**) Todd F. Elliott; (**d**, **h**–**j**) Rachel A. Koch; (**e**) Terry W. Henkel; (**f**) Stephen D. Russell; (**g**) Tatyana Svetasheva)

No single locus was sufficient to obtain a well-supported armillarioid phylogeny (see Additional file 2), but through the use of multiple loci, we were able to obtain a well-supported armillarioid phylogeny (Fig. 2). The six-gene analyses recovered a strongly supported armillarioid clade (100% BS and 1.00 BPP) (Fig. 2), which is composed of all known *Armillaria* and *Guyanagaster* species. The armillarioid clade is further composed of three well-supported lineages that were recovered from both analyses (Fig. 2): (1) the annulate lineage (100% BS and 1.00 BPP), which includes annulate mushroom-forming armillarioid species, (2) the exannulate lineage (91% BS and 1.00 BPP), composed of exannulate mushroom-forming armillarioid species formerly placed in *Armillaria* subgen. *Desarmillaria* and (3) the gasteroid lineage (100% BS and 1.00 BPP), composed of *Guyanagaster* species. The gasteroid lineage consists of two distinct species: *G. necrorhizus* from the Potaro region and an undescribed *Guyanagaster* species from the Mabura region of Guyana. These two species are geographically separated by mountains of elevations over 1000 m, as well as Kaieteur Falls and the Essequibo River, all within a distance of 125 km.

The annulate lineage is further composed of four well-supported lineages (Fig. 2), with species compositions as follows: (1) African lineage composed of *Armillaria fuscipes* and *A. camerunensis*; (2) melleioid lineage composed of *A. mellea*; (3) northern hemisphere lineage composed of *A. altimontana, A. borealis*, *A. calvescens*, *A. cepistipes*, *A. gallica, A. gemina*, *A. sinapina* and *A. solidipes*; and (4) Australasian + temperate South American lineage composed of *A. affinis, A. fumosa*, *A. hinnulea*, *A. limonea, A. luteobubalina, A. novae-zelandiae*, *A. pallidula* and *A. puiggarii*.

The most glaring conflict between single gene and multi-gene analyses is the placement of the African lineage: in *gpd*, *actin-1* and *EF1α* this lineage is nested within or sister to the Australasian + temperate South American lineage; in *TUB* and *RPB2,* it is sister to *A. mellea*; in 28S it is sister to *Guyanagaster*. In the multi-gene analyses, it is consistently recovered as diverging earlier than the *A. mellea* lineage. In all of the single gene analyses except for 28S, a close relationship between the exannulate and the gasteroid lineages was recovered.

### Age and ancestral range of *Armillaria* and *Guyanagaster*

**Fig. 3:**
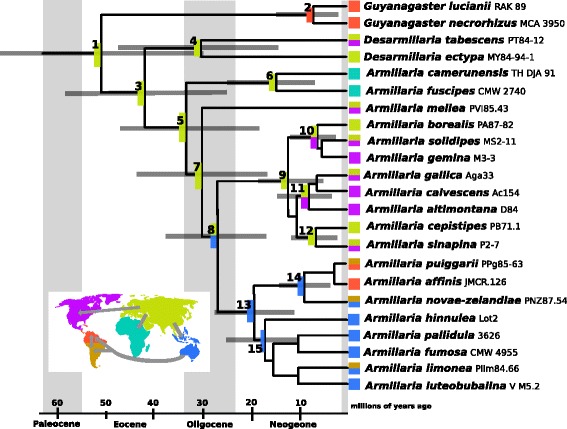
Time-calibrated phylogeny generated from Bayesian analysis of three gene regions (28S, *EF1α, RPB2*) from 19 *Armillaria* species, two *Desarmillaria* species and two *Guyanagaster* species. Geographic origin of each specimen is indicated by the box to the left of the name: *red* = Neotropics, *blue* = Australasia, *brown* = temperate South America, *lime green* = Eurasia, *purple* = North America, and *turquoise* = Africa. Boxes at ancestral nodes correspond to the most probable ancestral range at that node as presented in Additional file 4. Geologic epochs are noted above the time scale. Numbers at nodes correspond to lineages in Table 1 and Additional file 4. Dark grey bars correspond to the 95% HPD and correspond to the values in Table 1. The map in the lower left hand corner represents the proposed dispersal pattern

Table 1 has a complete list of the divergence dates for the well-supported nodes recovered in the phylogenetic analysis. Figure 3 is the time-calibrated phylogeny for the group. The time to most recent common ancestor (tMRCA) of the armillarioid clade estimated in the BEAST analysis was 51 Ma (node 1; 95% HPD 30–73 Ma). The tMRCA of the exannulate lineage was estimated at 41 Ma (node 4; 95% HPD 24–59 Ma). The tMRCA of the annulate armillarioid lineage was estimated at 33 Ma (node 5; 95% HPD 19–47 Ma). The date of the divergence between the two *Guyanagaster* species was estimated at 8 Ma (node 2; 95% HPD 3–14 Ma).

**Table 1:** Ages for nodes, confidence intervals and posterior probability on the phylogenetic tree presented in Fig. 3

Node	Lineage	Mean Age	95% HPD [min, max]	Posterior Probability
1	Armillarioid	50.81	30.03, 72.32	1.00
2	*Guyanagaster*	7.88	2.70, 13.91	1.00
3	*Desarmillaria* + *Armillaria*	41.32	24.47, 59.07	0.90
4	*Desarmillaria*	31.74	15.83, 47.71	1.00
5	*Armillaria*	33.00	18.90, 46.98	1.00
6	African lineage	15.49	6.84, 24.76	1.00
7	Melleioid lineage	30.12	17.73, 43.43	0.90
8	Northern hemisphere + Australasian + temperate South American lineage	27.52	17.13, 37.60	0.94
9	Northern hemisphere	12.07	6.12, 18.87	1.00
10	*A. borealis + A. solidipes + A. gemina*	6.66	2.41, 11.51	1.00
11	*A. altimontana + A. calvescens + A. gallica*	8.58	3.80, 14.02	1.00
12	*A. cepistipes + A. sinapina*	6.44	2.22, 11.24	0.52
13	Australasian + temperate South American	19.27	11.18, 27.99	0.98
14	*A. novae-zelandiae + A. affinis + A. puiggarii*	8.96	4.04, 14.47	1.00
15	*A. hinnulea + A. pallidula + A. fumosa + A. limonea + A. luteobubalina*	18.13	10.30, 26.29	--

We estimated the ancestral ranges for 15 well-supported clades. The most probable area was the same under range constraints of ≤ 2 and ≤ 3 for all nodes. The most probable ancestral range for the most recent common ancestor (MRCA) of the armillarioid clade is Eurasia. Eurasia is also the most probable ancestral range for all of the annulate mushroom-forming lineages containing northern hemisphere taxa. Migration to both Africa and Australasia from Eurasia is the most probable scenario as estimated from this analysis. Additional file 4 provides the probabilities for each area being the ancestral range under both area constraints; numbers in bold represent the most probable region at that node.

## Discussion

Our phylogenetic analyses, coupled with morphological data, support a three-genus composition of the armillarioid clade: *Armillaria* contains the annulate mushroom-forming species, *Desarmillaria* contains the exannulate mushroom-forming species, and *Guyanagaster* contains the known gasteroid species. Our estimates of the tMRCA of the armillarioid clade suggest it arose 51 Ma, which is in accord with a previous estimate [10], even though a different set of taxa, loci and calibration methods were used. During this time, Earth was experiencing one of the warmest intervals of the past 65 million years known as the Paleocene-Eocene Thermal Maximum (PETM) [55]. This warm and humid climate led to the widespread expansion of area covered in tropical rainforests. The ancestral range estimation suggests the most probable area for the origin of the MRCA is the Eurasian subcontinent (Additional file 4), much of which contained tropical rainforests at that time [56].

### Biogeography and evolution of *Guyanagaster* and *Desarmillaria*

Our phylogenetic reconstruction suggests that *Guyanagaster* species evolved within the earliest diverging lineage in the armillarioid clade. The process of gasteromycetation is believed to be unidirectional; gasteroid fungi evolve from a ballistosporic ancestor, and there is no evidence that this transformation has ever been reversed [11]. With this in mind, the ancestor to the armillarioid lineage was likely a gilled mushroom. According to our estimations, between 51 and 8 Ma the mushroom progenitor to *Guyanagaster* underwent the gasteromycetation process, resulting in the evolution of *Guyanagaster*. At one point in time, this ancient lineage was composed of mushroom-forming species—all of which likely went extinct—as the only known extant members of this lineage are *Guyanagaster* species.

The next lineage to diverge is composed of the two described *Desarmillaria* species: *D. ectypa* and *D. tabescens*. Neither of the species in this genus have an annulus at maturity, in contrast to all other known mushroom-forming armillarioid species. *Desarmillaria ectypa* is different from all other armillarioid species by virtue of its ecology: whereas *Armillaria* species are pathogens and wood decomposers, *D. ectypa* is restricted to peat bogs and associated with *Sphagnum* [57]. The ecology of *D. tabescens* is much more typical of *Armillaria* species. It is a primary pathogen towards introduced *Eucalyptus* species in France, as well as a secondary pathogen to *Quercus* species, but in many cases it is a saprotroph, colonizing stumps of *Quercus* species [58].

Both *Guyanagaster* and *Desarmillaria* evolved within the oldest lineages in the armillarioid clade and both genera contain two known species. *Armillaria* represents the most recently diverged genus-level lineage within the armillarioid clade, yet has over 35 described species (see [59] for species counts). The older ages of the lineages from which *Guyanagaster* and *Desarmillaria* evolved, coupled with the fact that they contain far fewer extant species, suggest these two genera are on a different evolutionary trajectory compared to *Armillaria*. As stated above, our ancestral range estimation suggests the most probable ancestral range for the MRCA to the armillarioid clade is Eurasia, whereas species of *Guyanagaster* have only so far been found in Guyana. One question is how the mushroom progenitor to *Guyanagaster* dispersed from Eurasia to Guyana. One possibility is that the mushroom progenitor to *Guyanagaster* was widely dispersed within the humid, tropical rainforest habitat that predominated at the time it arose. It is possible that *Guyanagaster* evolved within the Guiana Shield and represents a highly specialized endemic to this region.

The two species of *Desarmillaria* are thought to be more thermophilic than their sympatric *Armillaria* species [57, 60]. This temperature adaptation could be a residual characteristic of their origin during the PETM and could also explain the paucity of species in this genus. As the climate changed, species adapted for a cool and arid climate could have competitively displaced the species adapted for a warm and humid climate, in this case, species of *Desarmillaria* and the mushroom progenitor to *Guyanagaster*. The novel morphology of *Guyanagaster* species suggests that they are highly specialized for their current habitat, and as stated above, the restriction of *D. ectypa* to peat bogs is a specialized habitat for armillarioid fungi. Habitat specialization has been shown to act as buffer from extinction [61], and could be what has allowed species in these early diverging lineages to avoid competitive displacement by better-adapted armillarioid fungi. Whether *D. tabescens* occupies a specialized niche that has aided in its persistence to the present remains unknown.

### Biogeography and spread of *Armillaria*

According to our analyses, *Armillaria sensu stricto* started diversifying approximately 33 Ma in Eurasia, at which time the Earth’s climate shifted from the relatively ice-free world to one with glacial conditions; the tropical climate during which the MRCA of the armillarioid clade arose was replaced by a period of severe cooling and drying as well as an increase in seasonality [55]. This led to a contraction in the area covered by tropical rainforests [62] and the geographic expansion of species-poor deciduous vegetation [63]. Therefore, we hypothesize that *Armillaria* species represent annulate mushrooms that survived, diversified and radiated with their hosts as they adapted to the drier, cooler and seasonal climate (i.e. temperate) of the late Eocene and beyond.

*Armillaria* species in Africa form a monophyletic lineage (100% and 1.00 BPP). Single gene analyses and multi-gene analyses were ambiguous as to where this lineage diverged within the armillarioid clade (Additional file 2). In all multi-gene combinations, the African *Armillaria* lineage is resolved as the earliest diverging within *Armillaria sensu stricto*, which is similar to previous work that has also suggested that this lineage is divergent compared to the rest of the armillarioid lineage [10, 64]. Our ancestral area estimation suggests that the ancestral region of the African lineage is Eurasia. During the Eocene and Oligocene, a shallow seaway separated Africa from Eurasia, acting as a barrier to dispersal. In the early Miocene (19–12 Ma), the seaway drained, effectively linking the biotas in these two regions [65]. Our molecular clock analysis suggests that extant species in Africa started diversifying approximately 15 Ma, which fits a migration from Eurasia during this time.

Many northern hemisphere *Armillaria* species are found in both North America and Eurasia (see [58, 59]). In a study of *A. mellea*, which is a species distributed throughout the northern hemisphere, four distinct lineages were identified, corresponding to their locality: 1) western North America, 2) eastern North America, 3) western Eurasia and 4) eastern Eurasia [66]. They suggest that this species was once widespread and is now in the process of speciation [66]. Within this backdrop, it is possible that *Armillaria* species facilitated their dispersal as pathogens on the diverse flora that existed during this time. As available pathways disappeared, ultimately ceasing migration, the species started to diverge. From our ancestral range estimation, we can see that there were multiple armillarioid introductions into North America (Fig. 3), suggesting a dynamic process of dispersal across the northern hemisphere. A more comprehensive study on northern hemisphere *Armillaria* species is necessary to determine when and how they migrated between the major landmasses.

*Armillaria* species started diversifying in the temperate southern hemisphere (outside of Africa) about 19 Ma (Fig. 3, node 13). Our results suggest that the most probable dispersal pathway was from Eurasia to Australasia. In a plant biogeographic meta-analysis of the Australasian region, it was found that after 25 Ma, there was an increase of flora inputs to Australia from the IndoMalayan region, representing the time when the continental shelf containing Southeast Asia collided with that containing New Guinea and Australia [67]. The authors of this study hypothesized that habitat instability in the region at that time generated vacant niches and increased the probability of successful establishment of dispersed lineages compared with the relatively stable, saturated states existing prior to approximately 25 Ma [67]. In this scenario both the greater dispersal distance and relative difficulty in establishing viable populations together contributed to the infrequency of successful exchanges prior to 25 Ma. This too follows our hypothesis that *Armillaria* species dispersed as pathogens on flora that existed during this time.

Many of the same *Armillaria* species are found in both Australasia and temperate South America [68]. This pattern fits with an ancient Gondwanan distribution, but the lineage is far too young for this to be the case. Alternatively, the discovery of fossilized *Nothofagus* wood in Antarctic deposits from the Pliocene [69] suggests this could have been an overland dispersal route until 2 Ma. Many of the fungi in this Australasian + temperate South American lineage are thought to be close associates with *Nothofagus* [68]*,* suggesting again that they could have dispersed with their plant associates through Antarctica. Alternatively, long distance basidiospore dispersal events could account for the geographic disjunction in closely related *Armillaria* species. Because appropriate hosts—species of *Nothofagus—*occur in both Australasia and temperate South America [70], establishment after a long-distance basidiospore dispersal event could be more probable.

### The rise of rhizomorphs: morphological developments in the armillarioid clade

Our phylogenetic hypothesis sheds light on the evolution of rhizomorphs in the armillarioid clade. Rhizomorphs are discrete, filamentous aggregations that extend from a resource base into substrates that may not support their growth, foraging for new resource bases [71]. Individuals that produce rhizomorphs can occupy huge swaths of habitat and prevent other organisms from establishing, as is the case with the “humungous fungus” [72]. All armillarioid species have the capacity to form rhizomorphs in culture, but only the later diverging lineages have been observed to form them in nature (see Table 2, Fig. 1).

**Table 2:** Trophic strategy, rhizomorph production in nature and known geographic range of species used in this study

Species	Facultative Necrotroph?	Characterization	Rhizomorphs in nature?	Hosts	Known range	References
*Armillaria affinis*	Unknown		Unknown		Central America	[59]
*Armillaria altimontana*	Unknown		Yes	Hardwoods and conifers	Higher elevation forests of Western interior of North America	[83]
*Armillaria borealis*	Yes	Weakly pathogenic, opportunistic	Yes	Birch, wild cherry	Europe	[58, 84]
*Armillaria calvescens*	Yes	Opportunistic pathogen on stressed trees	Yes	Hardwoods, particularly sugar maple	Eastern North America	[85, 86]
*Armillaria camerunensis*	Unknown	Observed in a disease center, but unknown if it is the causal agent	None observed	Unknown	Africa	This study
*Armillaria cepistipes*	Yes	Weakly pathogenic	Yes	Conifers	Europe, North America	[58, 87]
*Armillaria fumosa*	Unknown		Unknown		Australia	[59]
*Armillaria fuscipes*	Yes	Particularly pathogenic to exotic species	None observed	Pine forest plantations, *Acacia* and *Cordia* species	Africa, India	[74, 75, 88]
*Armillaria gallica*	Yes	Weakly or secondarily pathogenic	Yes	Hardwoods	Europe, North America, Asia	[58]
*Armillaria gemina*	Yes	Primary pathogen	Yes	Maples, beech Birch	Eastern North America	[86]
*Armillaria hinnulea*	Yes	Secondary pathogen	Yes	In wet sclerophyll forests	Australia, New Zealand	[89, 90]
*Armillaria limonea*	Yes	Pathogenic to pine seedlings (introduced tree)	Yes	Pine	Argentina, Chile, New Zealand	[68, 91, 92]
*Armillaria luteobubalina*	Yes	Primary pathogen in native forests	Yes	Eucalyptus	Australia, Tasmania	[68, 93–95]
*Armillaria mellea*	Yes	Highly pathogenic	Yes	Over 600 ornamentals, hardwood and orchard trees	Europe, North America, Asia	[58, 96]
*Armillaria novae-zelandiae*	Yes	Pathogenic to pine seedlings (introduced tree)	Yes	Pine	Argentina, Australia, Chile, New Zealand	[91, 92, 95]
*Armillaria pallidula*	Unknown		Unknown		Australia	[59]
*Armillaria puiggarii*	Unknown	Observed in a disease center, but unknown if it is the causal agent	Melanized rhizomorphs observed in the field	*Dicymbe* spp.	Argentina, Bolivia Caribbean, Guyana	[95] and this study
*Armillaria sinapina*	Yes	Weakly pathogenic	Yes	Conifers	North America, Japan	[97]
*Armillaria solidipes*	Yes	Highly pathogenic	Yes	Conifers	Cooler regions of North America, Europe, China	[58, 98]
*Desarmillaria ectypa*	Unknown	Saprotrophic on decaying peat moss	No	Sphagnum moss	Europe, Russia, Japan, China	[57]
*Desarmillaria tabescens*	Yes	Highly pathogenic	No	*Eucalyptus, Quercus*	Asia, Europe, North America	[58, 73]
*Guyanagaster lucianii*	Unknown	Only saprotrophic stage observed	No	*Eperua* spp.	Guyana	This study
*Guyanagaster necrorhizus*	Unknown	Only saprotrophic stage observed	No, but short non-melanized hyphal cords may be produced	*Dicymbe* spp.	Guyana	[27]

Species in the earliest diverging genera in the armillarioid clade, *Desarmillaria* and *Guyanagaster,* have not been observed to form rhizomorphs in nature, but they do produce unmelanized rhizomorphs in culture [27, 57, 58, 73] (Fig. 1). Rhizomorph production of *Armillaria* species from Africa, the next lineage to diverge, has been scantily observed in nature [74], but the production of melanized rhizomorphs does occur in culture [75]. The next lineage to diverge is that composed of *A. mellea*, which produces short-lived melanized rhizomorphs with limited growth in the soil [58]. The most species rich armillarioid lineage, the northern hemisphere and Australasian/temperate South America lineage, produce melanized rhizomorphs in nature (see Table 1).

The capacity of *Armillaria* species to produce melanized rhizomorphs in nature is thought to be an adaptation to harsh environments [71]. Melanized rhizomorphs can confer advantages like protection against other microbial competitors, translocation of resources, growth from a suitable resource base into an environment that may not support growth, as well as enhancement of inoculum potential [71]. Additionally, melanin is thought to promote longevity and survival of rhizomorphs within the soil [76], and has also been found to help other fungi survive in extreme environments [77]. Although all species in the armillarioid clade appear to retain the trait of rhizomorph production, only the most recently diverged appear to be adapted for melanized rhizomorph production in the current environment. In a controlled environment, it was found that only under high oxygen availability and near saturated moisture would *D. tabescens* produce melanized rhizomorphs [78]. It was concluded that these environmental conditions are sufficiently stringent in the climate of today, and could explain why *D. tabescens* has not been observed to form rhizomorphs in nature. It is possible that the production of melanized rhizomorphs could be a driver of diversification in *Armillaria* by conferring a competitive advantage to the species that produce them.

*Desarmillaria* species lack an annulus at maturity, whereas *Armillaria* species have a robust annulus at maturity. An annulus is the remnant of a partial veil that remains after the pileus expands. Partial veils protect the immature hymenium [79]. The development of a more protected hymenium could be an adaptation of *Armillaria* to drier, more unpredictable habitats that were common when this lineage began to diversify.

## Conclusions

Our analyses suggest that the armillarioid clade arose in Eurasia during the PETM, a time marked by a warm, tropical climate. Two lineages arose during this time: the earliest diverging lineage—which eventually led to the gasteroid genus *Guyanagaster*—and the exannulate mushroom-forming genus *Desarmillaria*. Besides being the oldest lineages in the armillarioid clade, they are also depauperate compared to *Armillaria. Armillaria* diverged after the shift to a cooler and more arid climate at the Eocene-Oligocene boundary. The production of melanized rhizomorphs in nature and the development of a protective partial veil could be adaptations that led to the subsequent dispersal and diversification of *Armillaria* in the much harsher, temperate climate. The success of *Armillaria* could have displaced now-extinct species in the exannulate and gasteroid lineages. *Guyanagaster* and *Desarmillaria* species have likely persisted to the present because they are highly specialized for their habitat.

### Formal taxonomic descriptions

***Desarmillaria*** **(Herink) R. A. Koch & Aime gen. et stat. nov.**

Basionym—*Armillaria* ss. Fries subgenus Desarmillaria Herink, Sympozium o václavce obecné (J. Hasek). 1972 September. Lesnicka fakulta VSZ Brno: 44. 1973.

Type—*Armillaria socialis* (DC. ex Fr.) Herink, Sympozium o václavce obecné (J. Hasek). 1972 September. Lesnicka fakulta VSZ Brno: 44. 1973.

*MycoBank number*—MB 819124 (genus).

*Description*—Basidiomata stipitate, usually in caespitose clusters on wood. *Pileus* convex to applanate, usually some shade of brown. *Lamellae* pallid, adnexed to adnate to subdecurrent with lamellulae. *Stipe* central, various colorations but often concolorous with cap, longitudinally striate, usually with fibrils on upper third. *Annulus* absent. *Basidiospores* ellipsoid to spherical, smooth, hyaline, inamyloid. *Basidia* clavate, four-sterigmate, hyaline. *Cheilocystidia* clavate, usually resembling basidioles. *Pileipellis* suprapellis composed of round, ellipsoid, cylindric, utriform, brown to hyaline, verrucose cells; subpellis composed of a layer of compact, shortened hyphae. Clamp connections absent. Rhizomorph production not observed in nature, while unmelanized production in culture has been observed. Saprotrophic to parasitic. Known only from the northern hemisphere.

*Commentary—Desarmillaria* includes mushroom-forming armillarioid species that lack an annulus. This difference in morphology led Singer [80, 81] to divide *Armillaria* (as *Armillariella*) into two sections based on the presence or absence of an annulus at maturity. Herink [82], then, recognized *Armillaria* as an annulate subgenus and *Desarmillaria* as an exannulate subgenus. Additionally, members of this genus have not been observed producing rhizomorphs in the field [57, 58], which is in contrast to most *Armillaria* species, most of which do produce rhizomorphs in the field (see Table 2). Rhizomorph production in nature appears to be a lost trait in this genus, as species are still able to form them in culture.

We have opted to elevate *Desarmillaria* to genus-level for two reasons. First, the absence of an annulus in *Desarmillaria* species and the presence of one in *Armillaria* species is a reliable characteristic to differentiate the two genera. Second, our phylogenetic and molecular clock analyses show *Desarmillaria* is on a separate evolutionary trajectory compared to *Armillaria*, meriting a separate genus.

***Desarmillaria tabescens*** **(Scop.) R. A. Koch & Aime comb. nov.**

Basionym—*Agaricus tabescens* Scop., Scopoli 1772, Flora Carniolica Plantas Corniolae Indigenas no. 1537: 446.

*MycoBank number*—MB 819125 (species).

***Desarmillaria ectypa*** **(Fr.) R. A. Koch & Aime comb. nov.**

Basionym—*Agaricus ectypus* Fr., Fries 1821, Syst. Mycol. 1: 108.

*MycoBank number*—MB 819126 (species).

***Guyanagaster lucianii*** **R. A. Koch & Aime sp. nov** ***.*** *—Holotype—*BRG 41292, *isotype*—PUL F2891 (Figs. 1 and 3b–h). Mabura Ecological Reserve, (5°10'09.26"N, 58°42'14.40"W), 25 December 2013. R. A. Koch 89.

*MycoBank number*—MB 815807 (species)*.*

*Representative DNA barcode—*RAK 89 (holotype), ITS—GenBank KU170950, 28S—GenBank KU170940, *EF1α—*GenBank KU289110.

*Etymology*—Lucianii = in honor of “Lucian” Edmund, a Patamona fungal parataxonomist, who discovered the type locality of *G. lucianii* and has since been invaluable in collecting *Guyanagaster* specimens in the field.

**Fig. 4:**
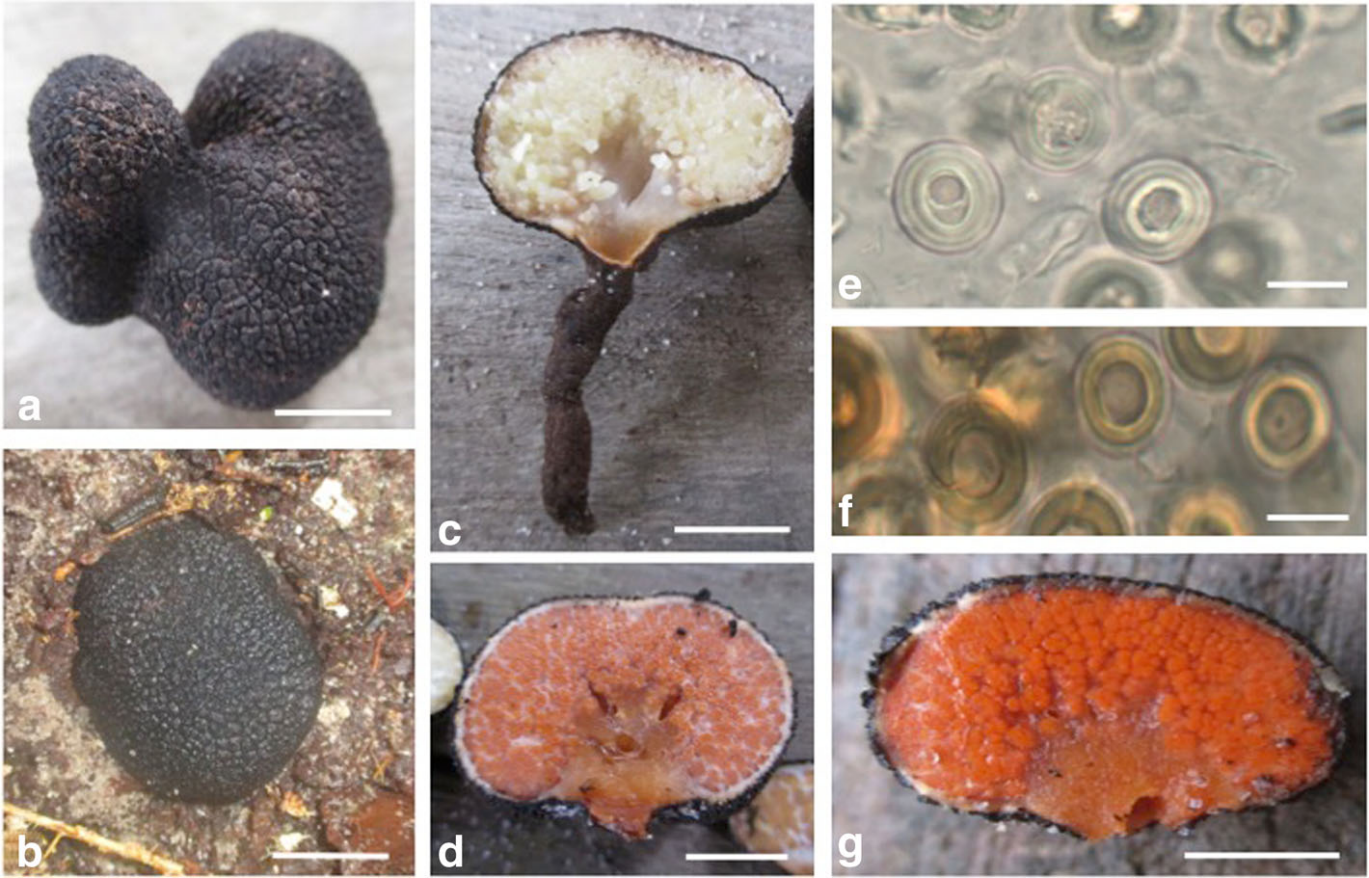
**a**–**g** *Guyanagaster lucianii* (BRG 41292 HOLOTYPE)*.* **a** Exterior showing dark brown peridium and irregularly lobed shape. **b** Subhypogeous habit. **c** Longitudinal section of basidioma showing immature gleba, developed columella and elongated stipe. **d** Maturing gleba. **e** Basidiospores of immature basidioma. **f** Basidiospores of mature basidioma. **g** Mature gleba. Bar = one cm in **a**, **b**, **c**, **d** and **g**. Bar = 20 μm in **e** and **f**

*Description*—Figures 2 and 4a–g. *Basidiomata* gasteroid, subhypogeous to hypogeous, scattered, or in linear troops, attached directly to woody roots of *Eperua falcata* and *Dicymbe altsonii* trees; 12–34 mm broad, 8–25 mm tall, globose to subglobose to ovoid and irregularly broadly lobate, dense, base with smooth, sterile concolorous stipe, 5–44 × 2–5 mm, attached directly to the substratum. *Peridium* dark brown (8 F4-8 F3) to black (8 F1) during all stages of development, moist, tough, covered in 4–6 sided polygonal pyramidal warts that come to a shallow point, these 0.8–3.1 mm broad, 0.5 mm tall, sharply differentiated from endoperidium; *endoperidium* white (8A1) to light pink (8A2), tough, 0.5–1 mm thick, composed of matted hyphae transitioning evenly to sterile, white hyphal veins between the glebal locules; *gleba* composed of well-defined locules and intervening veins of hyphae; locules globose to ovate to subangular, 0.2–2 mm broad, initially white (8A1), maturing in stages to gold (3A2-4A5), light orange-pink (7B6), to deep orange (8B8), often evenly, but rarely the locules that border the peridium and columella take on the darker shade first; hyphae separating locules initially white (8A1), hyaline and waterlogged in mature specimens; *columella* well-defined to not, base continuous with stipe, 6–13 × 3–6 mm, initially white (8A1), turning to brown (6D7), taking on the color of the gleba with age, cottony to gelatinous. *Exoperidium* individual hyphae of outer layer dark brown, individual hyphae curved to tortuously curved, thick-walled, non-gelatinous, 18–99 × 4–11 μm. *Endoperidium* 700–800 μm thick, well-differentiated from exoperidium and glebal locules, hyphae hyaline, 3–11 μm. *Sterile hyphae* separating locules similar to endoperidial hyphae, hyaline, thin-walled, branching, 3–5 um wide, organized in a parallel manner. *Basidia* not observed. *Basidiospores* globose, dextrinoid; 21.4–30.4 × 21.4–29.3 μm (mean = 25.1 ± 1.5 × 24.8 ± 1.5 μm, Q_r_ = 0.95–1.10, Q_m_ = 1.01 ± 0.04; n = 20); immature basidiospores hyaline when immature, then darkening to light pink and then rusty brown with maturity. *Columella hyphae* hyaline, thin-walled, not as tightly packed, some randomly inflated at septa, 4–13 μm wide, random apical cells inflated, 13–26 × 37–57 μm. *Clamp connections* absent in all tissues. *Taste* not tested. *Odor* not tested.

*Habit, habitat and distribution*—Attached to decaying woody roots of *Dicymbe altsonii* and *Eperua falcata* in tropical rainforests. Known only from the type locality in the Mabura Ecological Reserve of Guyana.

*Specimens examined*—*Guyana. Region 10 Upper Demerara-Berbice—*Mabura Hill, elevation 161 m; vicinity of basecamp, *Eduba* stand 4, four basidiomata, 11 June 2011, MCA 4424 (BRG 41294, PUL F3439); vicinity of basecamp, patch 32, one basidioma found at the base of a dead *Eperua falcata,* 24 December 2013, RAK 84 (BRG 41230, PUL F3440); vicinity of basecamp, patch 35, four basidiomata found at the base of a dead *Eperua falcata*, 24 December 2013, RAK 88 (BRG 41293, PUL F2892); 2.5 km north of basecamp, patch 31, nine basidiomata found along the decaying roots of *Eperua falcata*, 25 December 2013, RAK 89 (BRG 41292, PUL F2891).

*Commentary—* Phylogenetic evidence suggests that the two lineages of *Guyanagaster* represent distinct species that diverged approximately 8 Ma. The sister species share 87% (777/893 bp) nucleotide identity at the ITS region and 96% (705/731 bp) nucleotide identity at LSU. The drastic morphological changes that occur during the maturation of *Guyanagaster* basidiomata (i.e. gleba coloration) as well as the morphological plasticity between individual basidiomata (e.g. basidioma size and shape, columella dimensions) make delimiting species using macromorphological characters unreliable. However, *G. lucianii* can be distinguished from *G. necrorhizus* by its consistently larger basidiospores. During all stages of maturation, the basidiospores of *G. lucianii* measure between 21–30 × 21–29 μm, compared to those of *G. necrorhizus,* which measure 15–19 × 15–18 μm. Additionally, the basidiospores of *G. lucianii* lack the pedicel that is readily apparent on the basidiospores of *G. necrorhizus* (Fig. 4e–f).

Ecologically, *G. lucianii* appears to occupy the same niche as *G. necrorhizus*. Basidiomata are hypogeous to subhypogeous and growing from the roots of dead and decaying trees. Both *Guyanagaster* species are white rotters as the decaying roots to which they are attached are all light-colored, spongy and sometimes gelatinous. The known habitat for *G. necrorhizus* and *G. lucianii* are separated by only 125 km, so elucidation of their dispersal strategy is needed to understand the evolutionary forces that led to their speciation.

## Additional files

Additional file 1: Collection data for the specimens used ﻿in the phylogenetic analyses and GenBank accession numbers for the corresponding sequence data, both generated for this study and obtained from previous studies [10, 64, 83, 99–114]﻿. (XLSX 54 kb)
Additional file 2: Single-locus and multi-gene phylogenies for the six loci used. (PDF 3262 kb)
Additional file 3: Morphological description of *Armillaria camerunensis* TH DJA 91 and TH 9926. (DOCX 108 kb)
Additional file 4: Results from the ancestral range estimation analysis. (XLSX 38 kb)
