# Supplementary figures and images for: Resolved phylogeny and biogeography of the root pathogen Armillaria and its gasteroid relative, Guyanagaster
Source: BMC Evol Biol. 2017 Jan 25;17:33. doi: 10.1186/s12862-017-0877-3 (PMC5264464; doi:10.1186/s12862-017-0877-3)

# EF1a

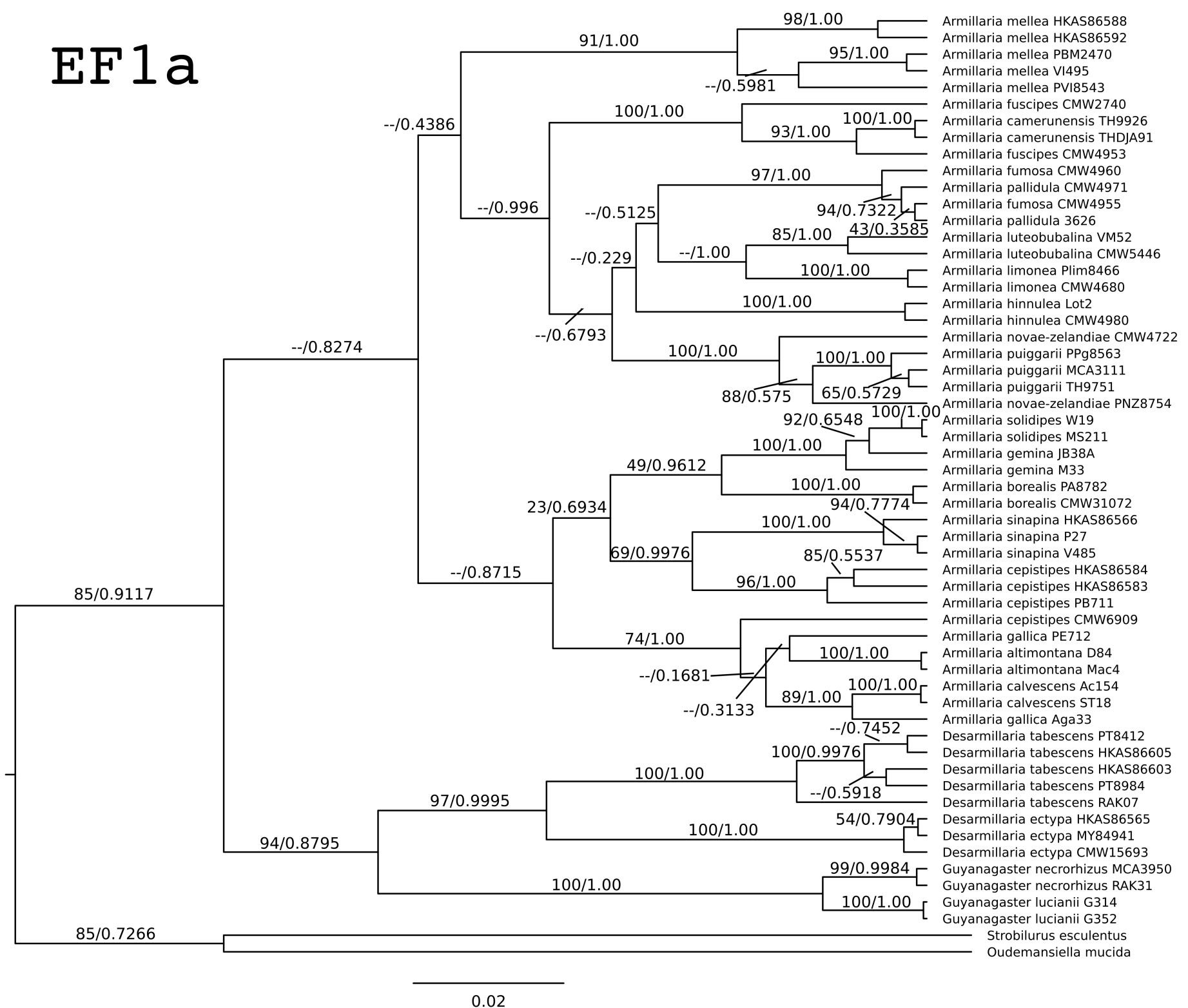

# RPB2

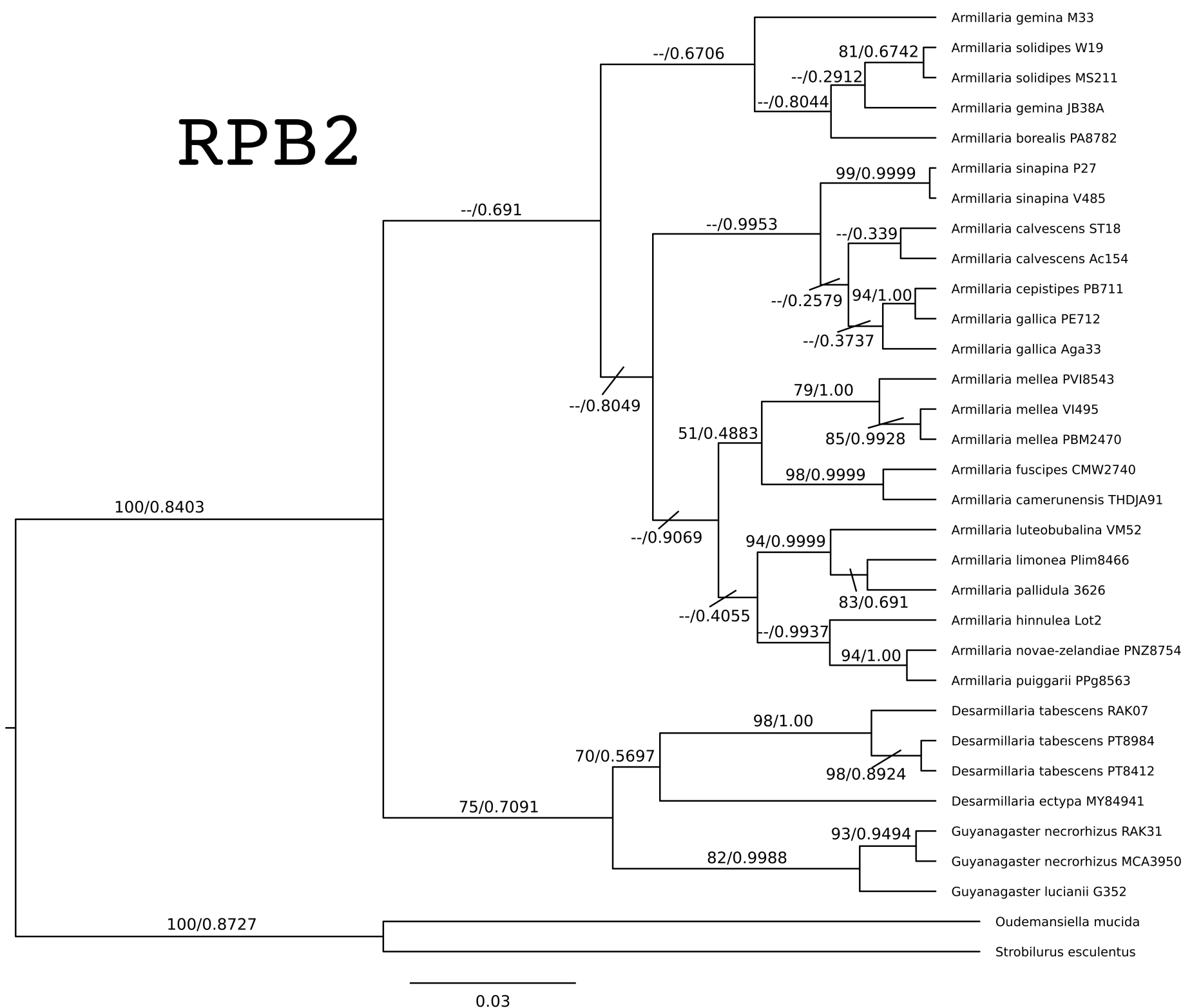

# Actin-1

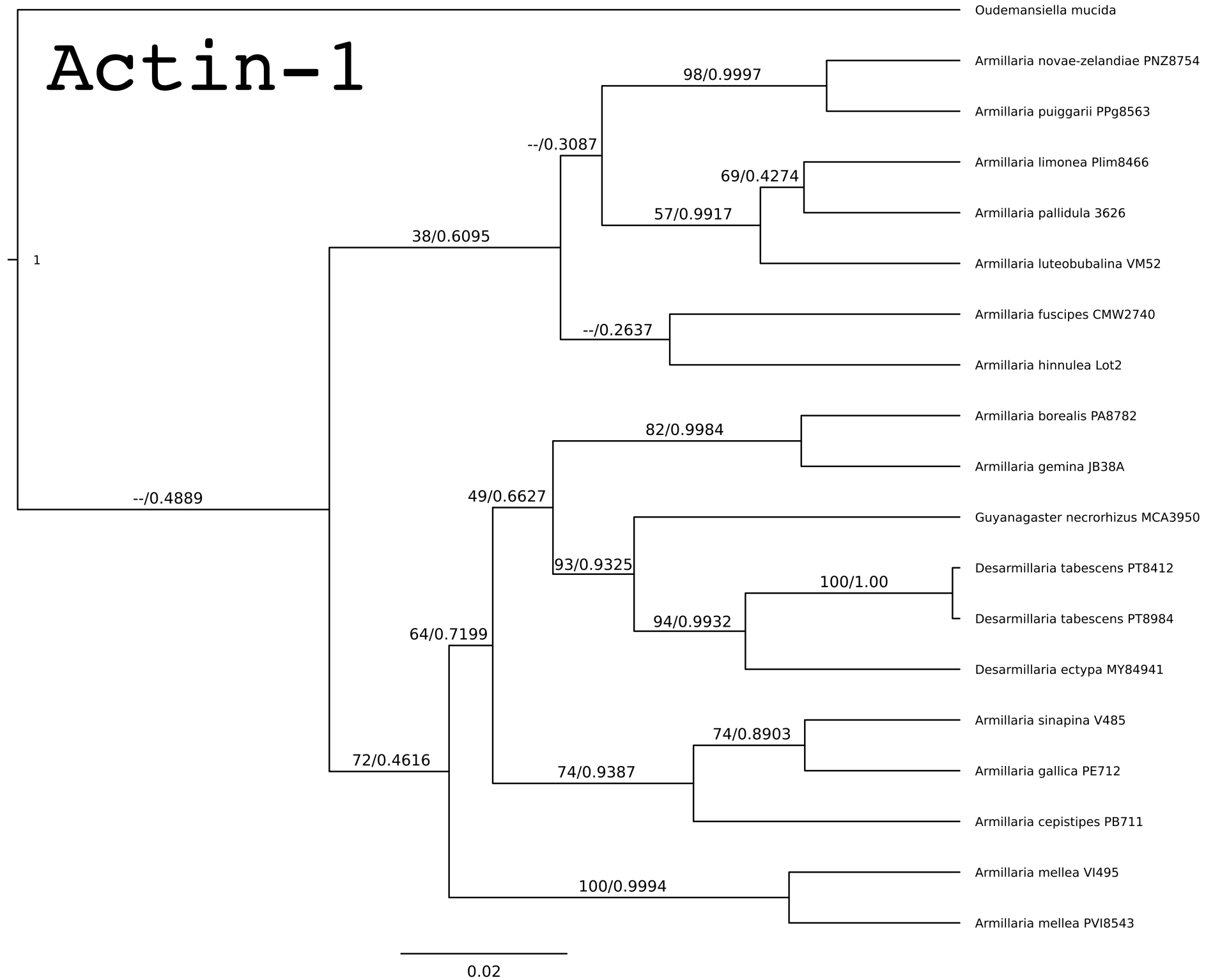

# 3GPD

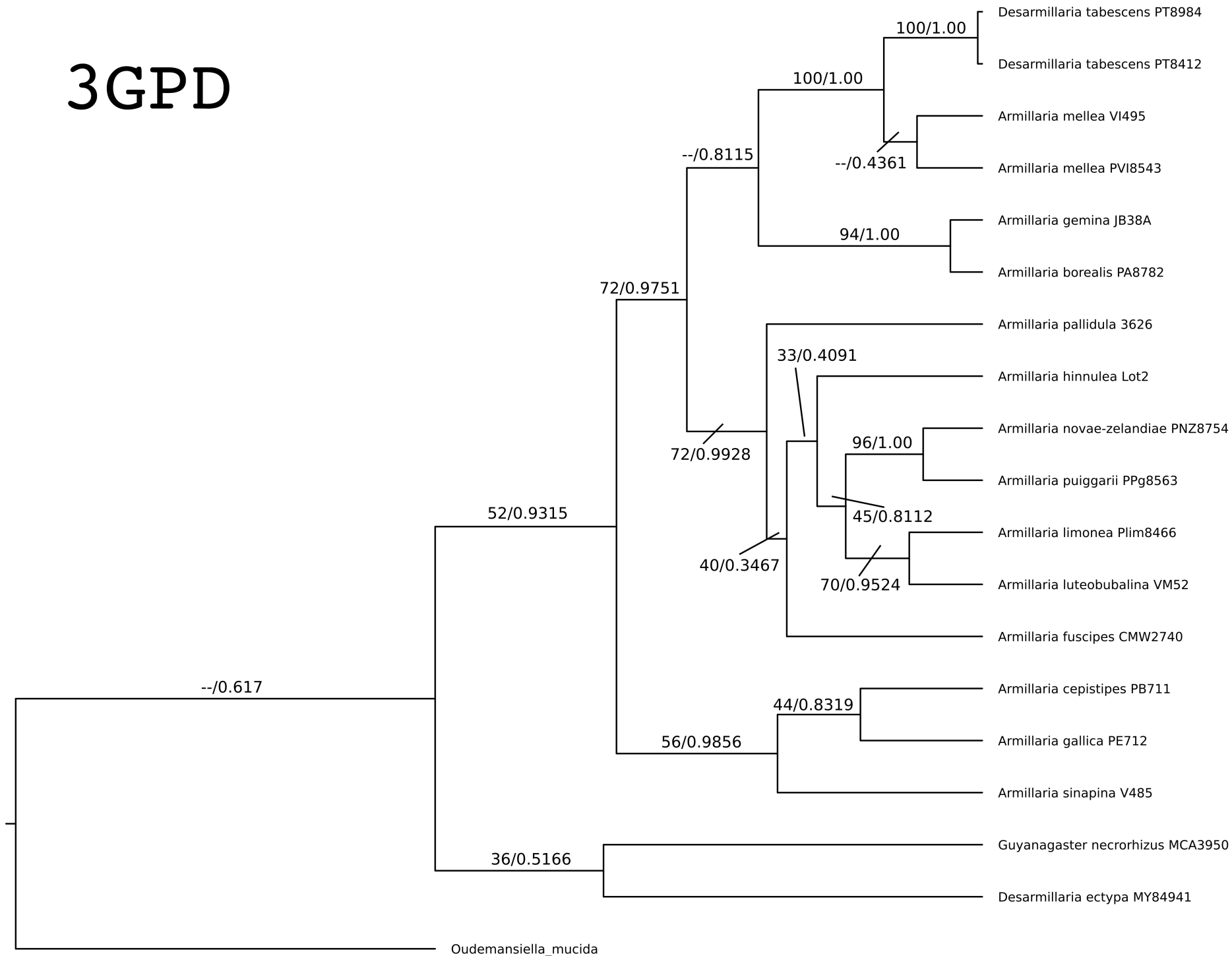

0.02

# TUB

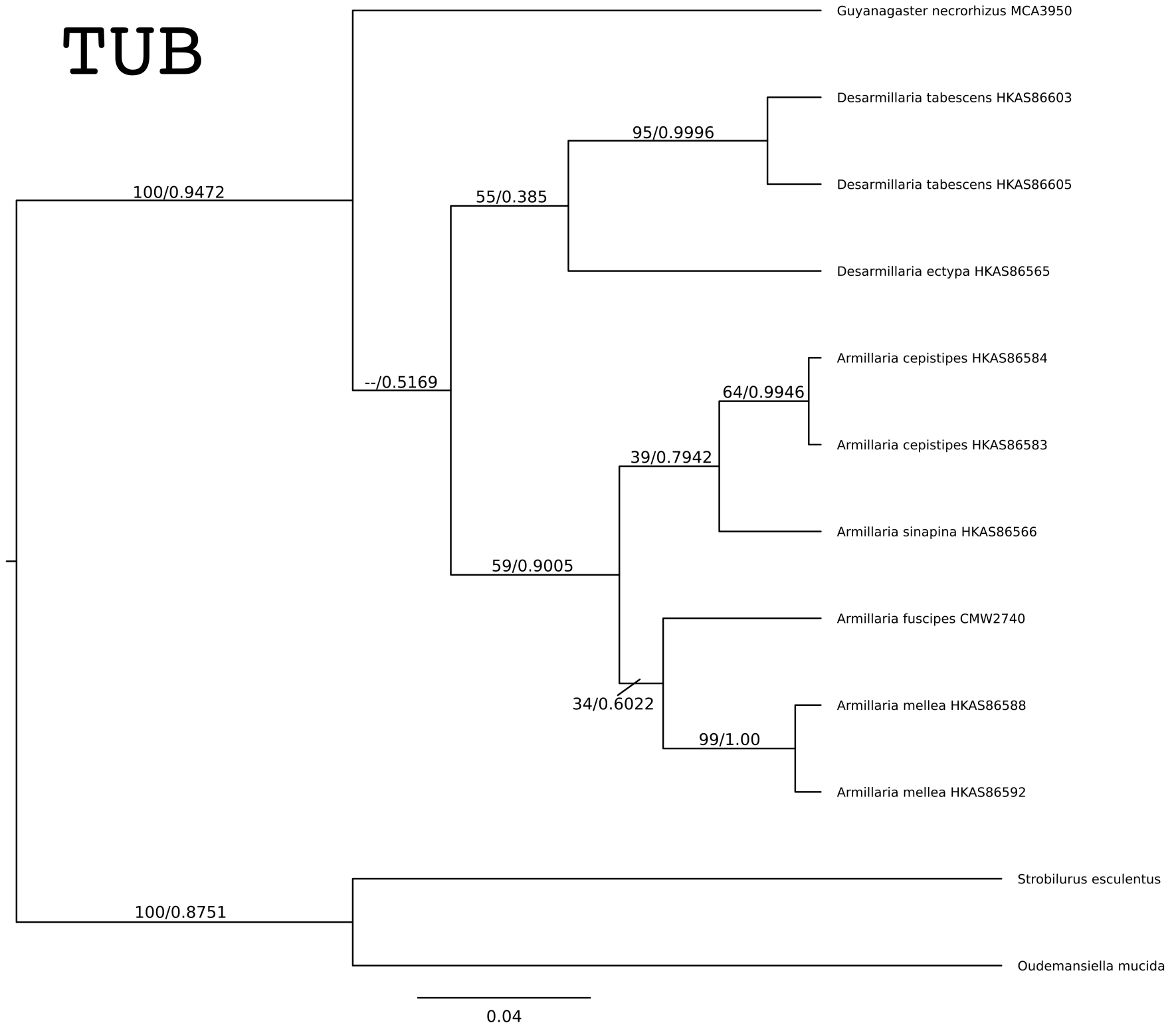

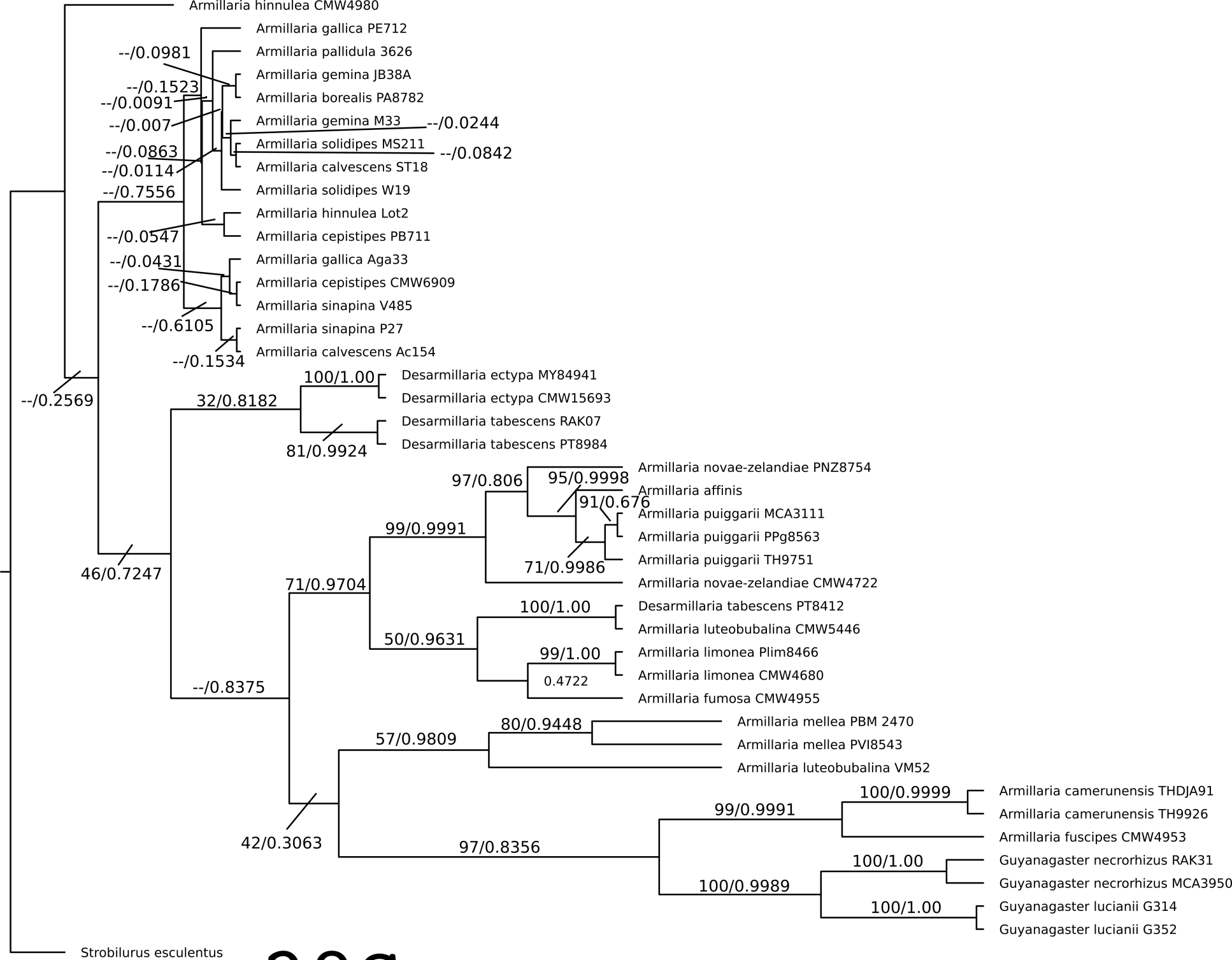

28S

0.005

0.5977

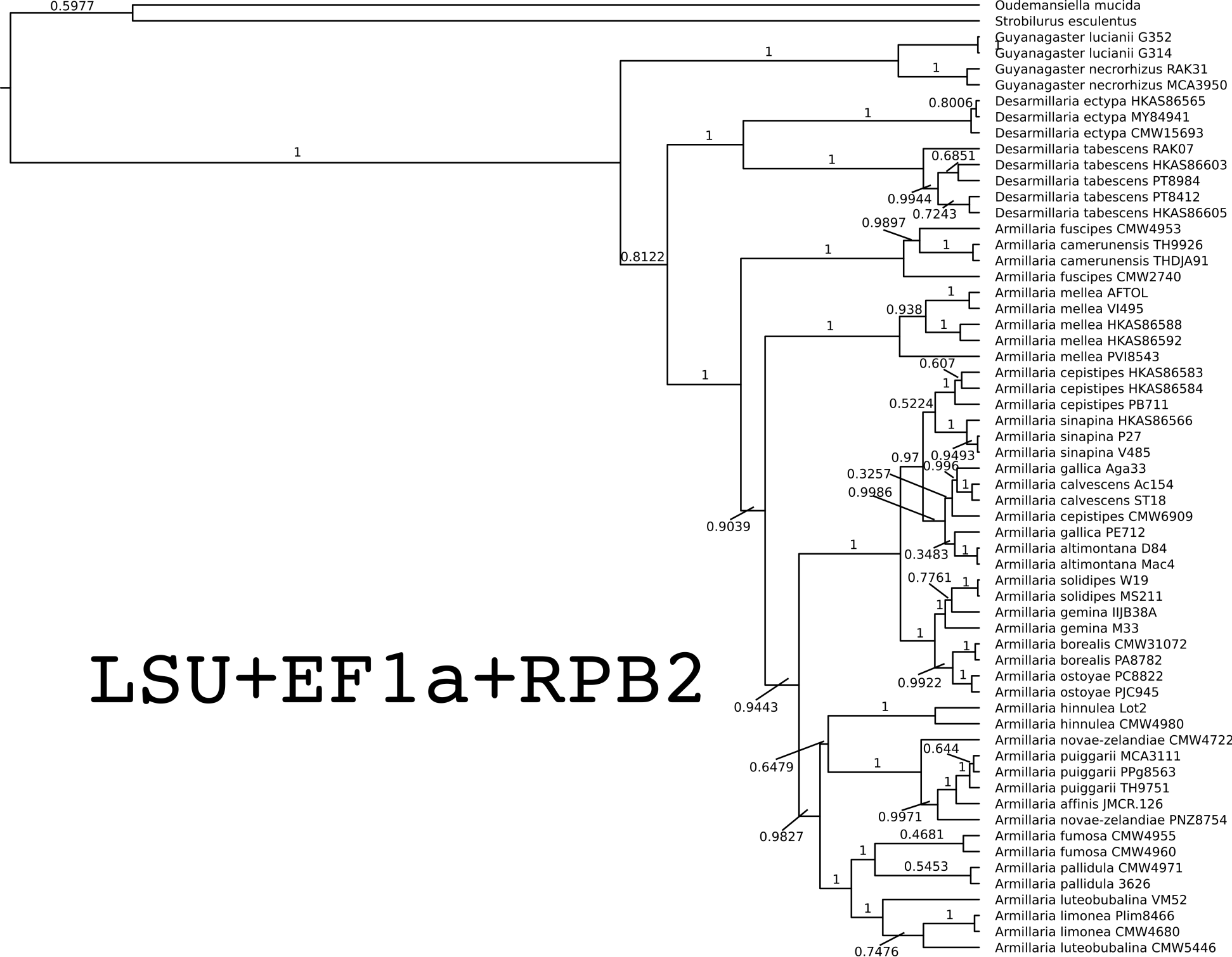

# EF1a+RPB2+TUB+actin1+3GPD

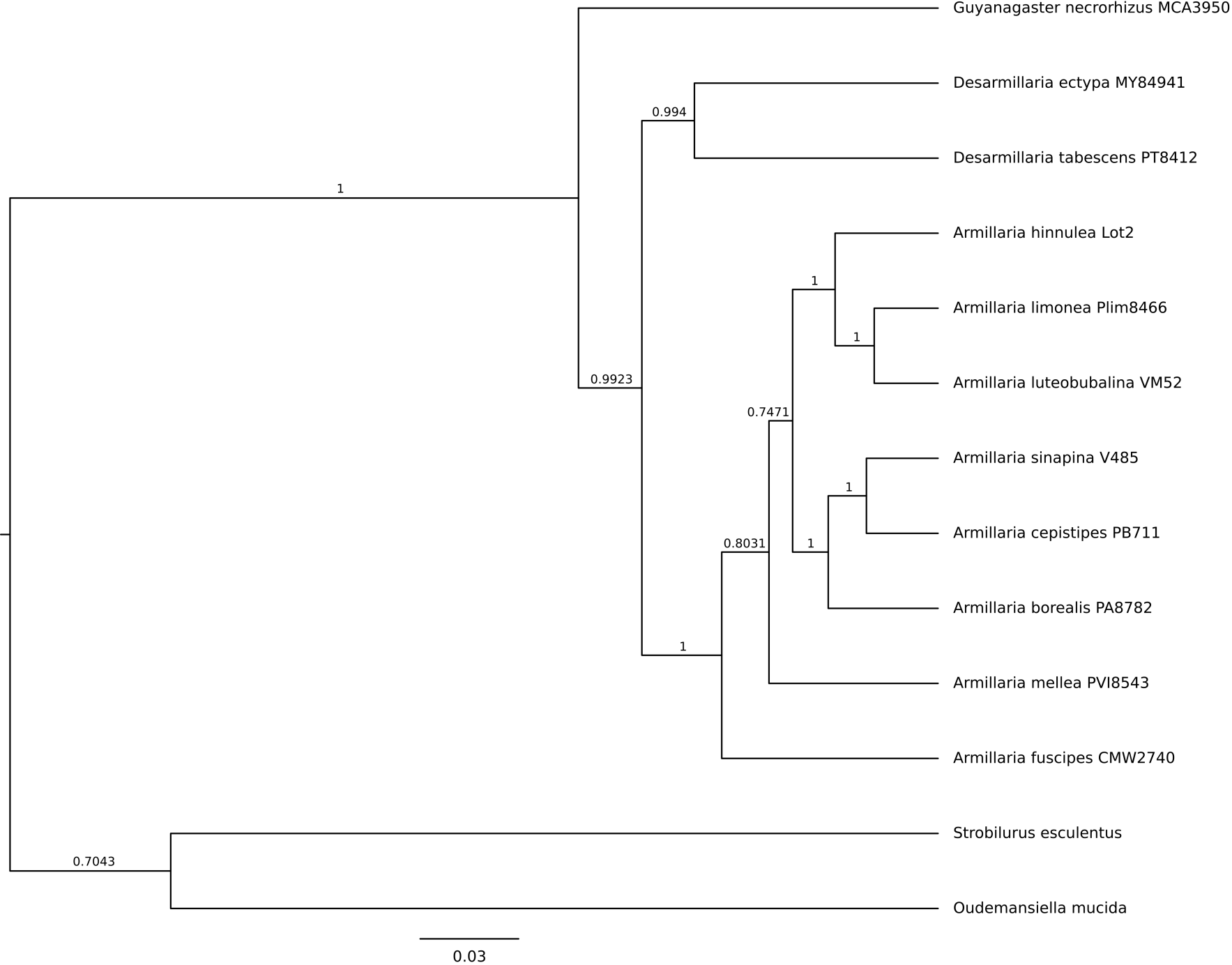

Supplement: Additional file 2: — Single-locus and multi-gene phylogenies for the six loci used. (PDF 3262 kb) [file 12862_2017_877_MOESM2_ESM.pdf]
