# Supplementary material for: Resolved phylogeny and biogeography of the root pathogen Armillaria and its gasteroid relative, Guyanagaster
Source: BMC Evol Biol. 2017 Jan 25;17:33. doi: 10.1186/s12862-017-0877-3 (PMC5264464; doi:10.1186/s12862-017-0877-3)
Supplement: Additional file 3: — Morphological description of Armillaria camerunensis TH DJA 91 and TH 9926. (DOCX 108 kb) [file 12862_2017_877_MOESM3_ESM.docx]

**Fresh Descriptions of *Armillaria camerunensis***

*Materials studied:* **TH DJA 91*—***PUL F2895, HSC G1182. Dja Biosphere Reserve, (3°21'29.8"N; 12°43'46.9"W), East Province, Cameroon, 5 September 2014. Collected by T. W. Henkel; **TH 9926**—PUL F2893. Dja Biosphere Reserve, (3°21'29.8"N; 12°43'46.9"W), East Province, Cameroon, 4 September 2014. Collected by T. W. Henkel.

*Representative DNA barcodes:* TH DJA 91: ITS—GenBank KU170952, 28S—GenBank KU170942, *EF1α—*GenBank KU289112; TH 9926: ITS—GenBank KU170851 , 28S—GenBank KU170941, *EF1α—*GenBank KU289111

*TH DJA 91 Fresh Description*—*Pileus* 35–60 mm wide, broadly convex with shallow central depression in larger specimens, central disc dark brown (6F8) becoming lighter brown (5C5-5C4) to almost cream at the margin (5A2-5A3), with erect, centrally concentrated, dark brown (6F8), fibrillose tufts becoming sparsely distributed on outer half, broadly striate on outer two-thirds, margin slightly upturned on mature specimens, otherwise entire, irregular to almost folded in larger specimens. *Pileus context* off-white, ~5 mm thick above stipe, thinning evenly to ~0.5 mm at margin. *Lamellae* adnexed, thick, crowded, not forking, cream (4A2-5A2), becoming light brown with age; edges entire, brown (5C5); *lamellulae* typically 3 per lamellae (one long in-between two short), occasionally 2 to 4, rarely up to 8, corresponding to marginal striations above. *Stipe* 16–65 × 5 mm, central, cylindric, even, dark grey-brown (8F3), becoming lighter towards the apex (5C4-5C5) longitudinally striate, upper third covered with erect fibrillose off-white tufts. *Annulus* well-developed, persistent, occurring 5–10 mm below stipe apex, protruding 2–3 mm from stipe, 1–5 mm thick, membranous, off-white, upper side densely tomentose, lower side covered with brown tufts, margin irregularly enrolled. *Stipe context* off-white, becoming darker towards base, solid. *Rhizomorph*s not observed. *Taste* not tested. *Odor* not tested.

*TH 9926 Fresh Description*—*Pileus* 10-15 mm wide, plano-convex, central disc brown (6F8) becoming lighter at the margin, beset throughout with erect somewhat recurved, sub-acuminate dark brown squamules concentrated over disc turning to sub-concentric rings over marginal two thirds, margin inrolled becoming incurved with maturity. *Pileus context* light brown (6B3-6B4), sub-solid, ~1 mm above stipe, thinning evenly to less than 0.5 mm at margin. *Lamellae* adnate with decurrent teeth, sub-thin to sub-thick, crowded, light flesh (6B3-6B4); edges concolorus with face, slightly roughened or occasionally brownish (perhaps due to damage); *lamellulae* 1-2, 1-1.5 mm long. *Stipe* 11-17 mm X 1.5-3 mm, central, cylindric, even, flesh cream (5A2-5A3), surface tomentullose under annulus with irregular, subaccuminate fibrous tufts beneath annulus, these becoming darker towards base, pulverulent above annulus. *Annulus* well-developed, occurring 2-3 mm below stipe apex, membranous, surface flesh tan, upper side white fimbriate, slightly flaring out. *Stipe context* concolorous, longitudinally fibrous with hollow central core. *Rhizomorphs* not observed. *Taste* indistinct, mildly fungoid. *Odor* none. Spore print *not obtained*.

*Micromorphology of TH DJA 91—Basidiospores* 6.7–9.0 × 5.2–6.4 μm (mean = 7.7 ± 0.6 × 6.0 ± 0.3 μm, Q_r_ = 1.1–1.6, Q_m_ = 1.3 ± 0.1; n = 20); inamyloid. *Basidia* 27.9–35.3 × 7.4–9.7 μm (mean = 31.7 ± 2.4 × 8.5 ± 0.6 μm; n = 20), clavate, four-sterigmate, sclerified; sterigmata 3–5 μm long (mean = 4.1 ± 0.4 μm), clamp connection absent at the base. *Cheilocystidia* resembling basidioles, abundant, 18–31 × 5–7 μm, mostly clavate, some lageniform, hyaline. *Pleurocystidia* not observed. *Pileipellis* consisting of a subpellis of hyphae 6–18 μm wide, thin-walled, branching, septate, hyaline, which blend into aggregations of brown to hyaline, verrucose, ellipsoid to clavate cells of the suprapellis, 20–100 × 8-35 μm, intermixed with filamentous units. *Stipitipellis* *hyphae* 5–22 μm wide, hyaline, septate. *Clamp connections* absent.

*Habit, habitat and distribution*—**TH DJA 91**: Fruiting on deadwood logs, on the floor of closed canopy lowland rainforest in the vicinity of *Uapaca* trees. **TH 9926**: Fruiting on medium size decaying log on ground from root ball tip-up with exposed decayed roots in monodominant stand of *Gilbertiodendron dewevrei*.
